# A Cross-Sectional Study on Oral Potentially Malignant Disorders: Diagnostic Challenges in Early Detection of Dysplasia and the Role of Velscope

**DOI:** 10.7759/cureus.69542

**Published:** 2024-09-16

**Authors:** Mounika Yeladandi, Umamaheswari Thirupambaram Nataraja Sundaram, Dhanya Muthukumaran

**Affiliations:** 1 Oral Medicine and Radiology, Saveetha Dental College and Hospitals, Saveetha Institute of Medical and Technical Sciences, Saveetha University, Chennai, IND

**Keywords:** dysplasia, fluorescence visualization, oral potentially malignant disorders (opmds), premalignant lesions, tobacco use, velscope

## Abstract

Background: The oral cancer burden has become a global challenge in the present scenario. Early diagnosis of oral potentially malignant disorders (OPMDs) with dysplasia is crucial to increasing the overall survival rate of the patients. Efficient chairside techniques are much needed to enhance diagnostic efficacy at early stages. This study evaluates the effectiveness of Velscope in detecting dysplastic lesions and analyzes the impact of lifestyle factors on the prevalence and progression of oral potentially malignant disorders among 40 participants.

Methods: A cross-sectional study was conducted with 40 participants diagnosed with OPMDs. Participants underwent a Velscope examination followed by incisional biopsy and histopathological evaluation. Data on lifestyle factors such as tobacco, alcohol, pan, and gutka use were collected through self-reported questionnaires. Data were analyzed to assess Velscope efficacy and correlations between lifestyle risk factors and lesion sites. Statistical analysis was performed using SPSS statistics for Windows (version 26.0, IBM Corp., Armonk, NY). The significance level was fixed at 5% (α=0.05).

Results: The data analysis of the study revealed that Velscope showed a sensitivity of 88.89% and a specificity of 46.15% (p = 0.013). The positive predictive value (PPV) of the Velscope was 77.42%, and the negative predictive value (NPV) was 66.67%. Overall, the diagnostic accuracy of the Velscope in this study was 75.0%. Cohen's kappa of 0.381 indicated moderate agreement with biopsy results. Biopsy results showed no statistically significant correlation was found between dysplasia and lesion site, gender, smoking, alcohol, or pan use except for gutka users, who had a significantly higher rate of dysplasia (p = 0.027).

Conclusion: Velscope demonstrated high sensitivity but moderate specificity in detecting dysplasia, emphasizing its role as an adjunctive tool in early detection and delineation of oral lesions in conjunction with biopsies for accurate diagnosis. The higher rate of positive results for dysplasia among gutka users suggests that focused public health interventions are needed to address significant lifestyle risk factors, which in turn could reduce malignant transformations in OPMDs.

## Introduction

According to Miranda-Filho and Bray, oral cancer accounted for 2.0% of all cancers and 1.9% of all cancer-related deaths, leading to the diagnosis of nearly 355,000 new cases and the associated death of over 177,000 people [1]. Although the five-year overall survival for oral cancer is about 50%, an early diagnosis can raise that to 85%. With survival rates closely correlated with the cancer stage at diagnosis, this bolsters the theory that early detection leads to better outcomes [2].

The world's highest incidence rates of oral cancer are in Asia, Europe, and Oceania. At the same time, Asia has the highest oral cancer mortality. The prevalence of attributable risk factors, such as alcohol consumption, betel quid use, and tobacco chewing and smoking, determines the incidence of oral cancer in populations. There is a higher incidence rate of six versus 2.3 per 100,000 people in the world age-standardised incidence rate, respectively, in males, who are culturally more exposed to the risk factors [3].

Given the protracted course and high risk of cancer associated with oral potentially malignant disorders (OPMDs), early detection and diagnosis of oral mucosal diseases are critical. Effective monitoring and intervention are essential for preventing oral cancer. The gold standards for investigating and assessing any questionable oral mucosal lesions include a thorough and methodical conventional oral examination (COE), prompt surgical biopsy, and histopathological evaluation [4,5]. However, this process heavily relies on the clinician’s subjective judgement and clinical experience, which may lead to missed or incorrect diagnoses. These challenges complicate the prevention and early detection of oral squamous cell carcinoma (OSCC) and OPMDs, as highlighted by Tomo et al. [6]. Therefore, more objective and efficient techniques are needed to enhance diagnostic accuracy.

When exposed to a light source with a specific wavelength range of 400-460 nm, naturally occurring autofluorescent substances in the normal oral mucosa and submucosa emit a green light known as autofluorescence [7]. The initial oral-use autofluorescence imaging device to receive approval was Velscope (LED Dental, Burnaby, British Columbia, Canada). The Velscope technology provides advantages such as non-invasiveness, quick usage, and user-friendly operation by utilizing tissue autofluorescence visualization technology. During detection, oral mucosal lesions are observed as black areas due to the loss of fluorescence visualization (FVL). This loss occurs due to the breakdown of substances that naturally emit fluorescence (autofluorescence substances) [8].

Significant variation in the diagnostic value of Velscope was found in a systematic review conducted by Cicciu et al. [8] on the clinical effectiveness of the instrument in the early detection of oral cancer and OPMDs. The range of sensitivity and specificity was found to be 8.4% to 100% and 22% to 100%, respectively. The average values for specificity and sensitivity were 65.95% and 70.19%, respectively. Autofluorescence can help non-specialists and less experienced professionals diagnose high-risk oral lesions, but these differences in diagnosis are influenced by operator skill and experience [9]. Low specificity in Velscope diagnosis was the main limitation in previous studies involving patients with OPMDs and/or OSCC [10].

Few studies have examined the use of Velscope in differentiating between high- and low-risk lesions in OPMDs, despite most studies focussing on the tool's usefulness in detecting oral cancer within OPMDs. Lesions on the ventral tongue or soft palate edges carry a significant risk of malignant transformation, although OPMDs are commonly found in the lining and masticatory mucosa [11]. This study analyzed subjective and objective autofluorescence examinations alongside histopathological results. Exploring Velscope's effectiveness in detecting cancer risk in OPMDs at different lesion sites is critical. The quantitative Velscope fluorescence method was employed to assess its diagnostic value, aiming to determine its suitability for detecting oral cancer given the varied reports on its diagnostic efficacy.

## Materials and methods

Study design and participants

This cross-sectional study included 40 patients clinically diagnosed with OPMDs during a screening camp in Hyderabad. The screening was conducted in collaboration with the Inner Wheel Club, District 315. This collaboration facilitated the organization of the camp, enabling community outreach and providing access to individuals for oral health screening. All the patients were examined with a Velscope, and the diagnoses were confirmed through histopathological examination following biopsy. The study followed ethical standards and received approval from the Institutional Review Board of Saveetha Dental College, Chennai, with the ethical number IHEC/SDC/PhD/OME-1908/24/084. The study protocol was explained to all participants, and written informed consent was obtained before initiating the study. The sample size for this study was calculated using the formula n=Z2*p*(1−p)/d2, where Z=1.96 (corresponding to a 95% confidence interval), p=0.0184 (prevalence of 1.84%), d=0.05 (the margin of error set at 5%), and the power of the study was set to be 80%. After obtaining the initial sample size, i.e., 32, a 10% adjustment was made to account for potential loss for any reason. The final sample size was determined to be 36, which is rounded off to 40 to achieve a representative assessment of Velscope’s diagnostic performance for OPMDs in our clinical setting [12].

Inclusion and exclusion criteria

The inclusion criteria for the study are adults aged 18 years and older who have been diagnosed with OPMDs, are willing to provide written informed consent, and have not undergone any previous treatment for their current oral lesion. Exclusion criteria for the study include patients below 18 years of age, those with a history of previous treatment for the current oral lesion, individuals with systemic diseases or conditions that may affect oral mucosal health, pregnant or lactating women, patients who decline to provide written informed consent, and those with lesions that cannot be properly visualized under Velscope due to anatomical constraints.

Endogenous fluorescence examination using Velscope

Patients underwent oral examination using the based device (LED Dental, Inc., White Rock, BC, Canada) before incisional biopsy. Examinations were conducted in a dark room, with patients wearing special glasses provided in the Velscope kit. A blue light (400-460 nm) was directed onto the oral tissues, and the emitted fluorescence was assessed visually. Initially, 10 patients were examined to calibrate the visualization of colours for consistent assessment across the study. Lesions exhibiting loss of autofluorescence (FVL) appeared as dark areas, suggesting dysplasia. After final surgical resection, tissue samples from the lesions were fixed in 10% formalin, stained with haematoxylin and eosin, and examined under a light microscope by an experienced oral pathologist. Magnifications of 10× and 40× were used to observe histopathological features of dysplasia like basal cell hyperplasia, nuclear hyperchromatism, cellular and nuclear pleomorphism, and altered nuclear-cytoplasmic ratio.

Statistical analysis 

Data analysis was performed using SPSS (IBM SPSS Statistics for Windows, version 26.0, Armonk, NY: IBM Corp., Released 2019). To evaluate the diagnostic performance of the Velscope device, several statistical measures were calculated by comparing the Velscope findings with the biopsy results.

Sensitivity and Specificity

The Velscope sensitivity, which measures its ability to identify true positive cases of dysplasia correctly, was determined. Likewise, the specificity, which measures the Velscope's ability to correctly identify true negative cases (i.e., nondysplastic cases), was also calculated.

Positive Predictive Value and Negative Predictive Value

The positive predictive value (PPV) represents the proportion of positive test results that are true positives, indicating the likelihood that a lesion identified as positive by the Velscope is indeed dysplastic. The negative predictive value (NPV) represents the proportion of negative test results that are true negatives, indicating the likelihood that a lesion identified as negative by the Velscope is not dysplastic.

Overall Accuracy

The Velscope overall accuracy was calculated by determining the proportion of true results (both true positives and true negatives) among the total number of cases examined.

Chi- Square Test and Fisher’s Exact Test

The chi-square test was used to compare the proportions of different diagnostic outcomes between groups. In cases where any expected cell frequency was less than five, Fisher’s exact test was employed instead, as it provides a more accurate significance level for small sample sizes.

Cohen’s Kappa

Cohen's kappa statistic assessed the agreement between the Velscope findings and biopsy results. This measure considers the possibility of agreement occurring by chance and provides a more robust evaluation of the consistency between the two diagnostic methods.

Roc Curve Analysis

Receiver operating characteristic (ROC) curve analysis was performed to evaluate the Velscope's overall diagnostic performance. The ROC curve plots the true positive rate (sensitivity) against the false positive rate (1-specificity) at various threshold settings, providing a comprehensive view of the test's ability to discriminate between positive and negative cases. The area under the ROC curve (AUC) was calculated to quantify the diagnostic accuracy, with a higher AUC indicating better diagnostic performance.

## Results

By employing comprehensive statistical analyses, the study aimed to rigorously assess the diagnostic value of Velscope in detecting malignant and potentially malignant oral lesions, thereby determining its efficacy as a diagnostic tool in clinical practice. The total sample size is 40, with 26 male and 14 female participants. The mean age of the male participants is 47.1 years with a standard deviation of 13.7, while the mean age of the female participants is 48.4 years with a standard deviation of 10.6 (Table 1).

**Table 1:** Demographic characteristics of the study participants This table shows the gender distribution of the study population with an overall mean age of 47.6 years and a standard deviation (Std. Dev.) of 12.6. N represents the number of participants.

Age parameters	Gender distribution		Total
	Male	Female	
N	26	14	40
Mean	47.1	48.4	47.6
Standard deviation	13.7	10.6	12.6

The Velscope identified 31 participants as positive for dysplasia. However, seven participants were false positives, leading to a specificity of 46.15% (95% CI: 23.21, 70.86). Additionally, the Velscope correctly identified 6 out of 13 participants as negative for dysplasia, yielding a NPV of 66.67% (95% CI: 35.42, 87.94). The PPV of the Velscope was 77.42% (95% CI: 60.19, 88.61), indicating that most positive results were true positives. Overall, the diagnostic accuracy of the Velscope in this study was 75.00% (95% CI: 59.81, 85.81), reflecting its effectiveness in detecting dysplastic lesions among the participants (Table 2).

**Table 2:** Estimation of efficacy of velscope with biopsy This table depicts the sensitivity, specificity, positive predictive value, negative predictive value and accuracy. CI: confidence interval, UB: upper boundary, LB: lower boundary.

Parameter	Estimate	95% CI (LB, UB)
Sensitivity	88.89%	(71.94, 96.15)
Specificity	46.15%	(23.21, 70.86)
PPV	77.42%	(60.19, 88.61)
NPV	66.67%	(35.42, 87.94)
Diagnostic accuracy	75.00%	(59.81, 85.81)

The biopsy results and Velscope findings agree moderately when categorized by fluorescence visualization patterns, according to Cohen's kappa of 0.381 and a statistically significant p-value of 0.013. Velscope found 7 negative samples and 24 positive samples (total = 31) under FVL and six negative samples and three positive samples (total = 9) under FVR. These findings suggest that Velscope, particularly its ability to detect fluorescence loss, may help diagnose and assess dysplasia in OPMDs, warranting further clinical investigation and integration into diagnostic protocols (Table 3).

**Table 3:** Comparison of biopsy results with Velscope findings This table shows that the results of Velscope findings are in agreement with biopsy results in terms of Cohen's kappa value and p-value.

Velscope findings		Biopsy			Cohen’s Kappa value	p-value
		Negative	Positive	Total		
Velscope	Negative	6	3	9	0.381	0.013
	Positive	7	24	31		
	Total	13	27	40		

Pan and tobacco chewing is a significant risk factor for oral health problems, including OPMDs. Gutka use, a form of chewing tobacco mixed with areca nuts and other ingredients, was reported by 17.5% of participants, while 82.5% did not. This type of tobacco is associated with an increased risk of oral lesions and cancers. Lastly, alcohol consumption was reported by 65.0% of participants, while 35.0% did not consume alcohol. These factors, when combined with tobacco use, can significantly increase the risk of oral cancers and related disorders (Table 4).

**Table 4:** Lifestyle factors of the study participants This table illustrates factors such as smoking, alcohol, gutka and pan, out of which a highest percentage of 72.5 was observed in patients with tobacco consumption.

Lifestyle factor	Category	Frequency	Percentage (%)
Smoking	No	29	72.5
	Yes	11	27.5
Tobacco	No	11	27.5
	Yes	29	72.5
Pan	No	34	85.0
	Yes	6	15.0
Gutka	No	33	82.5
	Yes	7	17.5
Alcohol	No	14	35.0
	Yes	26	65.0

Different oral cavity anatomical sites had different frequencies and percentages of lesions. Most lesions (55.0%) were in the left buccal mucosa (LBM). Then came right buccal mucosal (RBM) lesions (30.0%) and labial mucosa lesions (7.5%). Lip, tongue, and buccal vestibule lesions comprised 2.5% of cases (Table 5).

**Table 5:** Distribution of lesion sites This table shows results of 40 study participants with oral potentially malignant disorders, out of which 55% of lesions were present at the left buccal mucosa.

Site	Frequency	Percentage (%)
Left buccal mucosa	22	55.0
Right buccal mucosa	12	30.0
Labial mucosa	3	7.5
Lip	1	2.5
Tongue	1	2.5
Buccal vestibule	1	2.5
Total	40	100.0

Biopsy results were not associated with gender (p = 0.090), smoking (p = 0.999), tobacco use (p = 0.451), alcohol consumption (p = 0.999), or lesion site (p ranging from 0.027 to 0.160). However, gutka use was associated with dysplasia biopsy results (p = 0.027), suggesting that gutka users had a higher prevalence of positive biopsy results. These results highlight the complexity of risk factors for dysplasia in OPMDs and gutka use as a potential contributor requiring further study (Table 6).

**Table 6:** Comparison of biopsy results across categorical variables This table shows Fisher's exact p-values and categorical variables such as gender, risk habits, and site of the lesion. The significance level fixed was 5% (α=0.05).

Variable with sub categories		Biopsy						p-value
		Negative		Positive		Total		
		N	%	N	%	N	%	
Gender	Male	11	42.3	15	57.7	26	100.0	0.090*
	Female	2	14.3	12	85.7	14	100.0	
	Total	13	32.5	27	67.5	40	100.0	
Smoking	No	9	31.0	20	69.0	29	100.0	0.999*
	Yes	4	36.4	7	63.6	11	100.0	
	Total	13	32.5	27	67.5	40	100.0	
Tobacco	No	5	45.5	6	54.5	11	100.0	0.451*
	Yes	8	27.6	21	72.4	29	100.0	
	Total	13	32.5	27	67.5	40	100.0	
Pan	No	9	26.5	25	73.5	34	100.0	0.075*
	Yes	4	66.7	2	33.3	6	100.0	
	Total	13	32.5	27	67.5	40	100.0	
Gutka	No	8	24.2	25	75.8	33	100.0	0.027*
	Yes	5	71.4	2	28.6	7	100.0	
	Total	13	32.5	27	67.5	40	100.0	
Alcohol	No	5	35.7	9	64.3	14	100.0	0.999*
	Yes	8	30.8	18	69.2	26	100.0	
	Total	13	32.5	27	67.5	40	100.0	
Site	Left buccal mucosa	10	45.5	12	54.5	22	100.0	0.160*
	Right buccal mucosa	2	16.7	10	83.3	12	100.0	
	Labial mucosa	0	0	3	100.0	3	100.0	
	Lip	0	0	1	100.0	1	100.0	
	Tongue	1	100.0	0	0	1	100.0	
	Buccal vestibule	0	0	1	100.0	1	100.0	
	Total	13	32.5	27	67.5	40	100.0	

**Figure 1:**
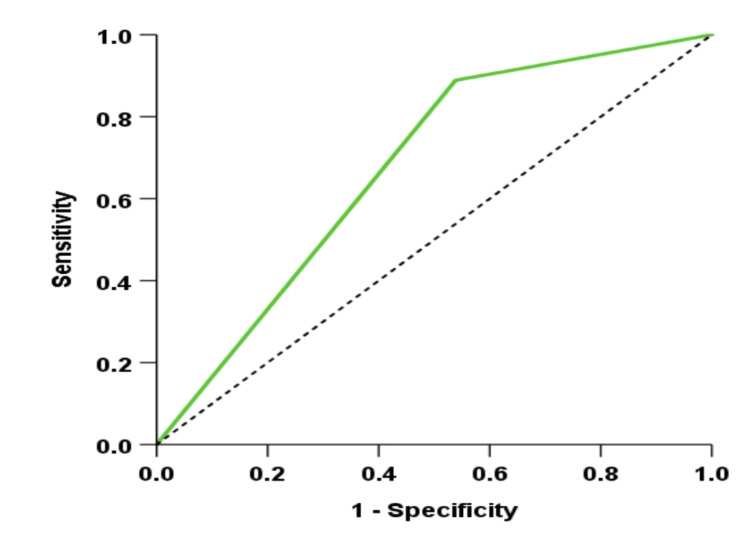
ROC curve The ROC curve, depicted by the green line, illustrates the relationship between sensitivity and specificity across different thresholds for Velscope.

The ROC curve demonstrates the diagnostic efficacy of the Velscope during the biopsy. The ROC curve, depicted by the green line, illustrates the relationship between sensitivity and specificity across different thresholds for Velscope. The proximity of the curve to the upper left corner indicates a high level of accuracy in detecting dysplastic or malignant changes. A higher area under the curve (AUC) with an area value of 0.675 suggests that the Velscope is effective in distinguishing between benign and dysplastic/malignant lesions, similar to the gold standard of biopsy (Figure 1).

## Discussion

Of the 40 participants in the study, 26 were male. The overall mean age of the participants is 47.6 years. The study's findings highlight the diagnostic performance of Velscope in detecting dysplasia in OPMDs among 40 participants, revealing both its strengths and limitations. The Velscope demonstrated a sensitivity of 88.89% and a specificity of 46.15%, with a PPV of 77.42% and a NPV of 66.67%. The overall diagnostic accuracy was 75.00%. These results suggest that while Velscope effectively identifies true positive cases of OPMDs, it has a moderate rate of false positives, as indicated by its lower specificity. These findings do not align with varying results from a few previous studies. For instance, a study by Al-Essa found Velscope to have a sensitivity of 74.1% and a specificity of 96.3%, demonstrating significant agreement with biopsy outcomes [13]. In contrast, Farah et al. reported lower sensitivity and specificity (30% and 63%, respectively), suggesting that while a Velscope can enhance lesion visibility, it should not be solely relied upon for definitive diagnosis without clinical interpretation [14].

The present study's high sensitivity indicates that Velscope is proficient at identifying individuals with OPMDs, which is crucial for early intervention and treatment. However, moderate specificity and the presence of false positives underscore the importance of using Velscope as an adjunctive tool rather than a standalone diagnostic method. The ability of a Velscope to highlight changes in tissue autofluorescence can aid in the early identification of pre-malignant and malignant lesions, thus potentially improving treatment outcomes and survival rates. The variability in Velscope's diagnostic performance across different studies can be attributed to several factors, including differences in study design, patient populations, and the types of lesions examined. While some studies report high specificity and PPV, others highlight the device's limitations in accurately identifying dysplasia, emphasizing the need for comprehensive clinical evaluation and corroborative diagnostic techniques such as biopsies [15].

The present study found that a high proportion of participants, 72.5%, admitted to using tobacco, which is a major risk factor for oral cancer and OPMDs. This finding is consistent with the study done by Zokaee et al., showing a strong correlation between tobacco use and the emergence of oral potentially malignant disorders such as leukoplakia and oral submucous fibrosis [16]. Additionally, the study found that 15% of participants reported using a pan, a chewing tobacco product linked to oral health issues and OPMDs. Furthermore, 17.5% of participants reported using gutka, a chewing tobacco product combined with areca nut, which has also been associated with a higher risk of cancers and oral lesions [17]. These findings emphasize the need for focused public health interventions, such as tobacco cessation programs and routine oral health screenings.

The LBM had 55.0% of lesions, the most common site. The RBM was second at 30.0% and the labial mucosa 7.5%. 2.5% of cases had tongue, lip, or buccal vestibule lesions. This distribution helps clinicians target screening, diagnosis, and management of OPMDs based on their common anatomical locations. These site-specific patterns must be understood for effective clinical management and early intervention to reduce malignant transformation risk. The site-specific pattern of the present study is consistent with several other studies. A study at the Amaltas Institute of Medical Sciences found that buccal mucosa was the most common site for oral premalignant lesions and conditions, including squamous cell carcinoma [18]. KIST Medical College and Teaching Hospital research found tongue lesions and tobacco-associated lesions were common, indicating the need to focus on those particular sites also to avoid missing such findings during oral examination [19]. A Chiang Mai University study found that most OPMDs, including lichen planus and leukoplakia, were found in the buccal mucosa and that smoking was strongly linked to dysplasia [20].

The biopsy results showed no statistically significant correlation between dysplasia and gender, lesion site, smoking, alcohol, pan. However, gutka users had a higher rate of dysplasia biopsy positives (p = 0.027). OPMD research emphasizes the role of several risk factors in their development, supporting this finding. Smoking has been linked to high-risk lesions, oral leukoplakia, a common OPMD, and cancer [21]. Alcohol and smoking are also risk factors for male OPMDs, according to other research. Not all of their effects on dysplasia are statistically significant [22]. Due to the prevalence of OPMDs like leukoplakia and erythroplakia and their correlation with dysplasia, especially in smokers, histopathological assessment is crucial [20]. Many non-plaque-induced gingival lesions, including premalignant lesions and conditions, have epithelial dysplasia, emphasizing the need for early detection and treatment [23]. The observed association between Gutka use and positive biopsy results for dysplasia suggests that targeted public health interventions are needed to reduce carcinogenic substance use and possibly reduce malignant transformations in OPMDs. These findings demonstrate the complexity of OPMDs and the importance of risk factor analysis in treating them.

Cohen's kappa of 0.381 indicates moderate agreement with biopsy results. Numerous studies support the effectiveness of fluorescence imaging in early oral lesion detection and tracking. The Velscope device uses blue light to detect tissue fluorescence, identifying visible and hidden oral lesions. This aids in oral cancer detection and may reduce mortality in cases where normal tissues lose collagen [24]. Recent advancements in ROC analysis have improved the calibration of diagnostic markers, enhancing the assessment of VELscope’s efficacy [25]. However, the predictive value of ROC curves may be misleading if not interpreted within the clinical context [26]. Thus, while the ROC curve and area under the curve (AUC) offer valuable insights into Velscope’s diagnostic capabilities, it is crucial to acknowledge their limitations and ensure that these metrics are integrated with clinical considerations for a more accurate evaluation of the device's performance. Research shows that Velscope can accurately define lesions that cannot be seen without assistance, which is crucial for surgically removing genetically abnormal cells. This is crucial for oral submucous fibrosis, which has the highest malignancy risk of any oral cavity disorder [27]. Autofluorescence and nuclear shape changes indicate severe disease. This implies that Velscope can identify mild dysplasia patients who require more intensive monitoring. Fluorescence imaging is useful for diagnosis, but it can give false positives in mild inflammation. It improves lesion margin assessment and complements white light examination.

The present study was carried out with a small sample collected from a screening camp conducted in one place. These add up to the limitations of the study, as the results cannot be generalized to the diversified population. Larger studies at different screening camps are suggested for validation.

## Conclusions

The findings from the current study underscore the diagnostic potential and limitations of Velscope in detecting dysplasia in OPMDs. With a sensitivity of 88.89%, Velscope effectively identifies true positive cases, which is crucial for early intervention and treatment. However, its moderate specificity of 46.15% indicates a significant rate of false positives, emphasizing the necessity of using Velscope as an adjunctive tool rather than a standalone diagnostic method. Additionally, a high proportion of participants using tobacco, pan, and gutka emphasizes the need for focused public health interventions, such as tobacco cessation programs and routine oral health screenings, to mitigate these risk factors. While the study found no statistically significant correlation between dysplasia severity and risk factors like tobacco and alcohol use, the higher rate of positive biopsy results for dysplasia among gutka users suggests targeted interventions could reduce malignant transformations in OPMDs. Overall, a Velscope is a valuable tool for early detection and delineation of oral lesions when used in conjunction with other diagnostic methods, contributing to better management of OPMDs and early-stage oral cancers.
